# Effectiveness of a multi-session combined near-peer and faculty-led surgical skills course on self-perceived ability to perform basic surgical skills

**DOI:** 10.1016/j.amsu.2020.07.045

**Published:** 2020-07-28

**Authors:** Billy Down, Simon Morris, Sagar Kulkarni, Kamran Mohiuddin

**Affiliations:** a Swansea University Medical School, Swansea University, Swansea, SA2 8PP, UK; b Morriston Hospital, Swansea, SA6 6NL, UK; c Medical Sciences Division, University of Oxford, John Radcliffe Hospital, Oxford, UK

**Keywords:** Medical students, Curriculum, Medical education, Surveys and questionnaires

## Abstract

**Background:**

Newly qualified UK doctors report feeling unprepared to perform basic practical procedures. The Royal College of Surgeons of England (RCSEng) responded to this concern by developing a national surgical curriculum, however, a national survey of UK medical schools identified that surgical skills teaching is inconsistent throughout the UK.

Peer assisted teaching sessions are delivered by senior students to junior peers and have been demonstrated to be effective in a number of settings. We aimed to develop a peer-led surgical skills course for medical students and assess its efficacy in teaching surgical skills.

**Methods:**

Combined near-peer and faculty-led teaching sessions were delivered to medical students (N = 14). We assessed for confidence in these skills using pre- and post-course Likert scale questionnaires to determine self-perceived benefit.

**Results:**

Overall, student confidence in all skills improved by +1.254 (p < 0.0001). Individually, confidence in every skill increased significantly, including continuous suturing, knot tying and excision and closure, which improved by +1.45 (p < 0.001), +1.22 (p < 0.05) and +1.59 (p < 0.0001), respectively.

**Discussion:**

This study demonstrates that teaching provided through near-peer faculty improves medical student confidence in basic surgical skills. A similar course design could be implemented in other UK medical schools to improve the surgical skills of newly graduated doctors

## Highlights

• This study shows an objective benefit to peer-led teaching surgical skills on undergraduate medical students' confidence.
• Near-peer learning is a beneficial process to students and teachers; equipping both with valuable skills and knowledge.
• Future medical curricula should consider incorporating near-peer style teaching in undergraduate surgical training. .

## 1 Introduction

Basic surgical skills are a core component of the undergraduate medical curriculum worldwide. There is widespread concern that newly qualified UK doctors are graduating with insufficient skills and training to safely perform basic practical procedures [1,2]. A national survey of UK medical schools identified that teaching of General Medical Council (GMC) mandated essential surgical skills was low; with 72.8% of schools teaching gowning and gloving, and only 24.7% teaching suturing [3].

With such inconsistency in the delivery of mandatory surgical skills teaching at medical school level, students may have to take it upon themselves to better their surgical skills [3]. Peer assisted learning is a novel technique in which teaching sessions are led by student's peers who may only be marginally more senior. It is based on the theory of cognitive congruence, where peer-teachers and their students share a similar knowledge base and learning experience, thus allowing peer-teachers to explain concepts at an appropriate level [4]. This technique has been demonstrated to be effective in the teaching of anatomy and surgical skills [5,6]. The benefits of peer-led sessions include: high tutor:student ratios, a relaxed atmosphere, and the ability to arrange more frequent sessions [7,8].

The sessions were designed with a constructivist approach; students were introduced to basic skills and subsequent tasks were designed with increasing complexity in order to embed the task in clinical practice and aid in application [9].

This paper aims to assess the benefit of a multi-session, near-peer led surgical skills course on students’ self-perceived confidence to perform surgical skills.

## 2 Methods

### Consent

Participants were informed that the data collected from anonymized questionnaires would be used to publish research data in scientific journals. Participants in agreement with data being used completed pre- and post-intervention questionnaires.

### 2.1 Participants and data sources

A four-part surgical skills course was delivered between December 2018 and March 2019 for undergraduate medical students at a single medical school. The course was advertised to all penultimate year medical students and participation was voluntary, however it was mandatory that students attend all four sessions if they signed up.

The near-peer teaching faculty consisted of two senior medical students, four junior doctors and a Consultant overseeing each session; with an average student to tutor ratio of 3:1. All tutors had completed accredited intercollegiate surgical skills training appropriate to their stage of training (including medical students).

The skills taught were based on GMC Guidelines in Outcomes for Graduates: Practical skills and procedures (2019), and the Royal College of Surgeons Basic Surgical Skills Course (Table 1) [9,10]. Sessions were taught with a constructivist approach: students were introduced to basic skills and subsequent advanced tasks were designed in order to embed the task in clinical practice. Students were taught skills based on Peyton's 4 step model [11]. Each skill was introduced via PowerPoint presentation, after which the students performed the skill on porcine tissue under supervision. The sessions' aims were outlined as follows:

- Session 1: Surgical instrument handling; hand ties; interrupted and continuous suturing.
- Session 2: Cyst excision and closure; subcuticular suturing.
- Session 3: Bowel anastomosis; arterial ligation.
- Session 4: Tendon repair (Kessler technique).

**Table 1:** Skills taught during surgical skills course.

Knot Tying Techniques	Suturing Techniques	Advanced Skills
Square	Interrupted	Cyst excision and closure
Surgeons	Continuous	Tendon repair
Aberdeen	Subcuticular	Artery ligation
At depth		Bowel anastomosis

Participants were asked to complete a questionnaire before and after attending each session. The questionnaires collected anonymized data on participants’ age, sex, year of study and previous surgical experience during medical school. Student confidence in each skill was assessed using Likert scales (1 [not confident at all], 2 [somewhat unconfident], 3 [neither confident nor unconfident], 4 [somewhat confident], 5 [very confident]). The students were also asked to rate the quality of the sessions using Likert scales (1 [strongly disagree], 2 [disagree], 3 [neither agree nor disagree], 4 [agree], 5 [strongly agree]). Data was compared between pre and post-session responses.

### 2.2 Analysis

Data were collated into Excel (Redmond, WA, USA) and analysed using GraphPad Prism 8 (La Jolla, CA, USA). T-tests (unpaired samples) to compare pre- and post-session mean confidence scores, and mean scores of responses to the audit questions were calculated.

## 3 Results

A total of 14 students participated and were included in the study.

Male to female ratio was 1.8:1. Average age was 26.77 years old (range 24–30).

**Fig. 1:**
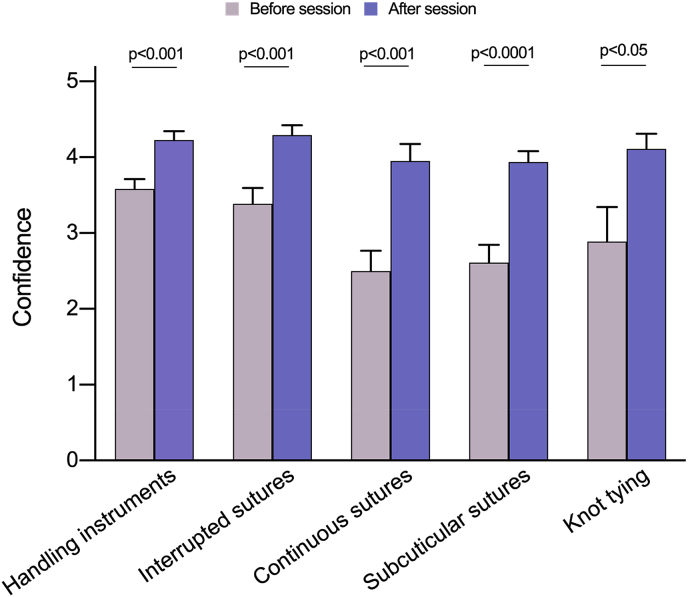
Pre- and post-session confidence in performing basic skills (instrument handling, knot tying and suturing).

Mean confidence improved significantly for all skills taught. Students reported improved confidence in al basic skills (Fig. 1): handling instruments (+0.65; p < 0.001), interrupted suturing (+0.9; p < 0.001), continuous suturing (+1.45; p < 0.001) and subcuticular suturing (+1.33; p < 0.0001), knot tying (+1.22; p < 0.05).

**Fig. 2:**
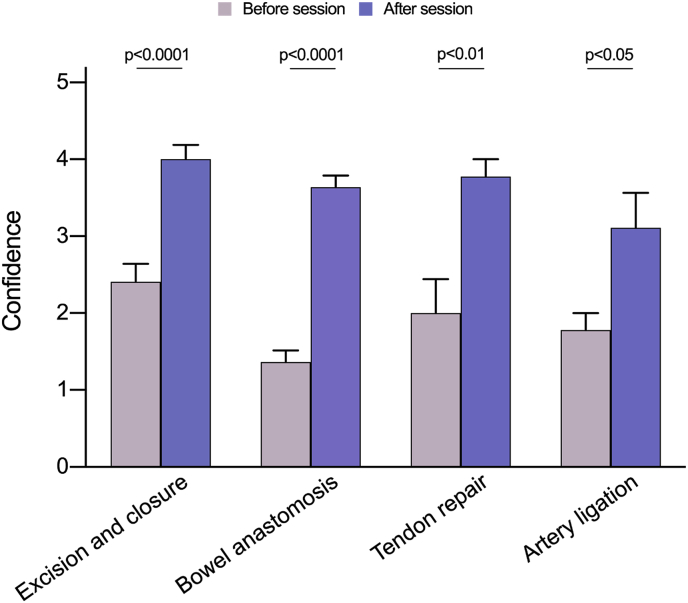
Pre- and post-session confidence in performing advanced surgical skills (tendon repair, bowel anastomosis, arterial ligation and cyst excision).

Furthermore, confidence was also improved for the advanced skillset (Fig. 2): excision and closure (+1.59; p < 0.0001), bowel anastomosis (+2.28; p < 0.0001), tendon repair (+1.78; p < 0.01) and artery ligation (+1.33; p < 0.05).

Prior to the course, 100% (n = 14) students had scrubbed into surgery, 92.8% (n = 13) had handled instruments and 14.3% (n = 2) had sutured.

On a scale of 1–5 (1 = not comfortable at all, 5 = very comfortable), students responded to “how comfortable do you feel in theatre?” with a mean pre-course score of 4.15 and a post-course score of 4.46 demonstrating a numerical increase in comfort level, although it did not reach statistical significance. 100% (n = 14) of students felt that the course had clear objectives and was pitched at an appropriate level.

## 4 Discussion

The Royal College of Surgeons has acknowledged concerns in the variability of surgical skills teaching throughout the UK by developing a national undergraduate curriculum in surgery [12]. The curriculum refers to essential procedures as mandated by the GMC which include: use of local anaesthetics, wound care, skin suturing and surgical asepsis. The proportion of medical schools providing this teaching is low, leading to many students graduating feeling underprepared for their foundation years [3]. A recent national survey demonstrated that only 48% of medical students felt prepared to participate in an acute surgical take versus 79% for an acute medical take [13].

This study assessed the benefit of a surgical skills course taught by a near-peer faculty ranging from senior medical students to junior doctors with Consultant oversight. The primary aim was to improve student's confidence in performing basic skills and ensure that students would feel comfortable in future theatre attachments. The results showed that prior to the course, only 2/14 students had performed any surgical skill beyond scrubbing and handling instruments. Student confidence significantly increased in performing all basic surgical skills, and the course was well received, scoring almost maximum points in all areas of feedback. The authors recognise that the advanced skills taught are far beyond that expected of a medical student. However, these tasks were designed with a constructivist basis, where each session built on the previous schema and allowed student to embed their new knowledge in clinical application; thus aiming to maximize retention [14]. This was reflected in the feedback and post-course confidence of students.

The modern medical curriculum focusses heavily on the development of transferable qualities such as communication, ethics and research [15]. Basic surgical skills such as skin suturing and knot tying are transferrable to many specialties including surgery, emergency medicine and even general practice with many general practitioners (GPs) developing special interests in minor surgery. Therefore, understanding the role of a surgeon and the experience of a patient within the operating theatre should be a requirement for all doctors in order to fully engage with their patients.

The operating theatre can be an intimidating environment for students, especially those not armed with the fundamental knowledge of theatre etiquette and basic surgical skills. This can lead to students avoiding the theatre environment, rather than actively pursuing it [16,17]. In addition, with increasing time pressures facing the NHS, it is not uncommon for theatre lists to be over-booked and thus limit the time available intra-operatively for training and teaching medical students and junior doctors alike [18]. This may have detrimental effects on the number of students pursuing a career in surgery.

The value of near-peer learning allows students to be taught by junior medical staff in a more relaxed and less intimidating environment which is likely to increase student uptake of opportunities. High tutor:student ratios allow for effective hands-on teaching and the potential for development of lasting relationships beneficial to both student and teacher [7]. The limitation is that skills may not be taught accurately, however the authors of this study attempted to overcome this by ensuring that each session had consultant oversight. Furthermore, the tutor is able to develop their teaching skills while also relieving the burden on time-pressured senior faculty members. The provision of near-peer learning sessions may allow more time to be spent with students learning skills, which is essential in surgery, and allow teaching to be delivered earlier in student's undergraduate careers.

Knowledge and skill retention occurs with repeated exposure to teaching and practice [19]. Our study has demonstrated that a surgical skills course provided by a multi-modal faculty of individuals from near-peer to consultant over a 4 month period is an effective means of teaching medical students. The mixed level faculty delivers a combination of strengths, providing high tutor:student ratios, a relaxed informal atmosphere, a combination of expert experience from consultants and empathy from junior trainees, the opportunity to develop friendships with potential future mentors, and the ability to run sessions frequently due to the high number of faculty members. We urge other university surgical societies to consider offering extra-curricular surgical training to medical students.

The course received very strong feedback in all areas from the students. We attribute this to the qualities discussed above, and we also provided high quality animal tissue (Wetlab Ltd) to provide high fidelity scenarios, and a spiral curriculum with skills repeated over several sessions to allow students to learn and then consolidate skills.

### 4.1 Limitations

Although the small sample size (n = 14) limits the conclusions that can be drawn from this pilot study, this is the first mixed level faculty taught course in the UK to our knowledge. There was a likely selection bias as the students who applied were more likely to be already interested in a career in surgery. We sought to minimise this by making the application process very simple and not setting any pre-requisites. The course was carried out in a graduate-entry program with average ages above the national average, and this may make results less transferrable to other medical schools.

## 5 Conclusion

All medical schools in the UK provide Bachelor of Surgery to all graduates yet the lack of surgical training during undergraduate education is well recognised. This study suggests that small interactive surgical skills sessions led locally by a faculty combining a range of career grades leads to significantly increased confidence in performing surgical skills, with a number of added benefits possibly unique to a mixed level faculty.

This study shows an objective benefit to peer-led teaching surgical skills on undergraduate confidence. Future medical curricula should consider the incorporation of near-peer learning in core surgical modules.

## Funding

Nil.

## Ethical Approval

Nil required.

## Consent

Consent gained (see article). All data anonymous.

## Registration of Research Studies

1 Name of the registry:
2 Unique Identifying number or registration ID:
3 Hyperlink to your specific registration (must be publicly accessible and will be checked):

## Provenance and peer review

Not commissioned, externally peer reviewed.

## Guarantor

Billy Down.

## CRediT authorship contribution statement

**Billy Down:** Conceptualization, Writing - original draft, Writing - review & editing. **Simon Morris:** Writing - original draft, Writing - review & editing. **Sagar Kulkarni:** Writing - original draft, Writing - review & editing. **Kamran Mohiuddin:** Supervision, Writing - review & editing.

## Declaration of competing interest

None.
